# Host Transcription Profile in Nasal Epithelium and Whole Blood of Hospitalized Children Under 2 Years of Age With Respiratory Syncytial Virus Infection

**DOI:** 10.1093/infdis/jix519

**Published:** 2017-09-27

**Authors:** Lien Anh Ha Do, Johann Pellet, H Rogier van Doorn, Anh Tuan Tran, Bach Hue Nguyen, Thi Thu Loan Tran, Quynh Huong Tran, Quoc Bao Vo, Nguyen Anh Tran Dac, Hong Nhien Trinh, Thi Thanh Hai Nguyen, Bao Tinh Le Binh, Huu Mai Khanh Nguyen, Minh Tien Nguyen, Quang Tung Thai, Thanh Vu Vo, Ngoc Quang Minh Ngo, Thi Kim Huyen Dang, Ngoc Huong Cao, Thu Van Tran, Lu Viet Ho, Bertrand De Meulder, Charles Auffray, Jorrit-Jan Hofstra, Jeremy Farrar, Juliet E Bryant, Menno de Jong, Martin L Hibberd

**Affiliations:** 1 Oxford University Clinical Research Unit, Wellcome Trust Major Overseas Program, in partnership with the Hospital for Tropical Diseases, Ho Chi Minh City, Vietnam; 2 Murdoch Children’s Research Institute, Melbourne, Australia; 3 European Institute for Systems Biology and Medicine, Lyon, France; 4 Centre for Tropical Medicine and Global Health, Nuffield Department of Clinical Medicine, University of Oxford, United Kingdom; 5 Children Hospital 1, Ho Chi Minh City, Vietnam; 6 Children Hospital 2, Ho Chi Minh City, Vietnam; 7 Department of Medical Microbiology, Academic Medical Center, University of Amsterdam, The Netherlands; 8 Genome Institute of Singapore; 9 London School of Hygiene & Tropical Medicine, United Kingdom

**Keywords:** children under 2 years old, host expression profile, lower respiratory tract infections, respiratory syncytial virus, rhinovirus

## Abstract

**Background:**

Most insights into the cascade of immune events after acute respiratory syncytial virus (RSV) infection have been obtained from animal experiments or in vitro models.

**Methods:**

In this study, we investigated host gene expression profiles in nasopharyngeal (NP) swabs and whole blood samples during natural RSV and rhinovirus (hRV) infection (acute versus early recovery phase) in 83 hospitalized patients <2 years old with lower respiratory tract infections.

**Results:**

Respiratory syncytial virus infection induced strong and persistent innate immune responses including interferon signaling and pathways related to chemokine/cytokine signaling in both compartments. Interferon-α/β, NOTCH1 signaling pathways and potential biomarkers HIST1H4E, IL7R, ISG15 in NP samples, or BCL6, HIST2H2AC, CCNA1 in blood are leading pathways and hub genes that were associated with both RSV load and severity. The observed RSV-induced gene expression patterns did not differ significantly in NP swab and blood specimens. In contrast, hRV infection did not as strongly induce expression of innate immunity pathways, and significant differences were observed between NP swab and blood specimens.

**Conclusions:**

We conclude that RSV induced strong and persistent innate immune responses and that RSV severity may be related to development of T follicular helper cells and antiviral inflammatory sequelae derived from high activation of BCL6.

## Teaser

RSV infection induces a clearly different host response pattern compared with hRV and induced strong innate immune responses both locally and systemically. B cell lymphoma (BCL6) is a hub gene that positively correlates with RSV load and disease severity.

Respiratory syncytial virus (RSV) is the leading cause of lower respiratory tract infections (LRTIs) in young children. Although some risk factors associated with severe RSV disease have been identified (prematurity, underlying lung or heart diseases, immunodeficiencies), the majority of children with RSV that require hospitalization were previously healthy and lacked comorbidities [1, 2]. The pathogenesis of RSV remains poorly understood, and no vaccines are available. Microarray techniques provide an excellent hypothesis-generating tool to explore the expression patterns of transcripts across entire biological pathways during the course of disease [3]. The advent of microarray technologies enables investigating the host factors involved in the immediate response to infection and those that modulate RSV disease severity. Several RSV host expression profiling studies have been conducted using in vitro models [4–10], animal models [11–15], and human subjects [16–22]. However, most in vivo studies only investigated systemic transcriptional profiles in blood [17–19, 21, 22], with only 1 study exploring local respiratory expression profiles by analysis of nasopharyngeal (NP) aspirates of hospitalized children (n = 30) [23], and 1 study, using an animal model, exploring overlapping components of respiratory and systemic responses [15]. Because the burden of RSV disease is greatest amongst children <2, and because patterns of RSV pathogenesis in young children are likely to differ significantly from immune responses of adults or animal models, specimens from pediatric cohorts represent a priority for in-depth analysis.

In this study, we applied microarray technology to acute and early recovery blood and NP swabs obtained from children <2 years hospitalized for LRTI with laboratory-confirmed RSV or rhinovirus (hRV) infections, which was reported as a second pathogen found in this population [24].

Using a combination of weighted gene coexpression network analysis (WGCNA) [25] and conventional analysis approaches [26], this unique data set allowed us to compare local and systemic host responses with RSV and hRV infections and to correlate these to clinical outcomes to identify key genes involved in RSV pathogenesis and severity.

## Materials and methods

### Ethics

The following Institutional Review Boards approved the study: Children’s Hospitals (CH)1 and 2 and the Hospital for Tropical Diseases (Ho Chi Minh City, Vietnam) and the Oxford University Tropical Research Ethics Committee (Oxford, UK). Written informed consent was obtained from parents or legal guardians of children before enrollment into the study.

### Study Design, Setting, and Population

The clinical data and samples for gene expression profiling were collected from a previously reported cohort of 632 children with LRTI under 2 years of age hospitalized at CH1 and CH2 [24]. Severe cases were defined as being hospitalized in the intensive care unit or requiring supplemental oxygen/mechanical ventilation or having a peripheral capillary oxygen saturation (SpO2) <92%. In brief, at admission (acute phase) and discharge or day 7 of hospitalization (early recovery), blood was collected in ethylenediaminetetraacetic acid (EDTA), and PAXgene ribonucleic acid (RNA) tubes (PreAnalytiX, Hombrechtikon, Switzerland) and 2 NP swabs (Copan Diagnostics Inc, Murrieta, CA) were collected in RNAlater (Sigma, Singapore) and viral transport medium (VTM) [27]. Nasopharyngeal swabs in RNAlater and blood in PAXgene RNA tubes were processed for host gene expression profile analyses. The EDTA tubes and the NP swabs in VTM were processed for blood count and detection of 14 respiratory viruses, including RSV and hRV [28–30]. Based on the results of the viral detection on NP swabs at admission, and the availability of paired samples, 343 cases were selected and classified into 3 groups for the current study: single-RSV group (RSVsi) (n = 177 of 343, 52%), RSV coinfection group (RSVco) (n = 88 of 343, 26%), and single-hRV group (hRV) (n = 78 of 343, 22%) (Supplementary Figure 1). The RSV group includes patients from RSVsi and RSVco.

### Gene Expression Profiling by Microarray

Total RNAs from whole blood and NP swabs were extracted using the PAXgene blood RNA isolation kit (PreAnalytiX) and the RNeasy Mini kit (QIAGEN, Hilden, Germany), respectively. Biotinylated amplified complementary RNA (cRNA) was generated by in vitro transcription of total RNA using Ambion Illumina TotalPrepRNA amplification kit (Ambion Inc., Austin, TX), according to the manufacturer’s instructions. After purification, 800 ng of cRNA from NP samples and 2000-ng labeled cRNA from blood were hybridized to the HumanHT-12 v4 BeadChip array at 55°C for 18 hours and under washing, blocking, and streptavidine-Cy3 staining steps, according to the manufacturer’s instructions. Each array on the HumanHT-12 v4 Expression BeadChip targets more than 31 000 annotated genes with 47 231 probes derived from the National Center for Biotechnology Information Reference Sequence RefSeq Release 38 (November 7, 2009) and other sources. The array was scanned using an iScan confocal scanner (Illumina Inc., San Diego, CA).

### Preprocessing and Quality Control of Microarray Data

Using R statistical software version 3.2.1 (R Development Core Team) [31], raw expression data files from the Illumina iScan system were preprocessed using Bioconductor lumi package [32] for quality control, background correction, transformation, and normalization (robust spline normalization algorithm). The ArrayQualityMetric package [33] was used for summarizing quality of metrics data and detecting outlier arrays that differed substantially from other arrays in the dataset [34]. If an array from paired samples of a given patient was removed, this patient and all associated data were also removed from the final analysis. Based on the quality control analysis, only 83 cases were processed for the final microarray analysis (4 arrays from every case, 332 arrays), RSVsi (n = 38 of 83, 46%), RSVco (n = 15 of 332, 18%), and hRV (n = 30 of 83, 36%) (Supplementary Figure 1 and Supplementary Table 1). After normalization and filtering, the expression data of NP and blood consisted of transcript levels for 30 020 and 24 228 probes, respectively, being processed for downstream analysis of differentially expressed genes (DEGs) and WGCNA. Gene expression data are available at the GEO database (https://www.ncbi.nlm.nih.gov/geo/query/acc.cgi?acc=GSE97743) under accession number GSE97743.

### Identification of Differentially Expressed Genes

To investigate differences in gene expression between 2 time points (early recovery versus acute phase) for each group (RSV, RSVsi, RSVco, and hRV), the linear modeling framework in the limma package [26] was used. A DEG was defined by a fold change ≥2 or ≤−2 and Benjamini-Hochberg false discovery rate corrected *P* < .05. Due to the design of Illumina BeadArray, there were multiple probes for each gene, reflecting the possibility of multiple differentially spliced transcripts for each gene. We collapsed this information as such that each probe represented an average transcript for each gene.

### Weighted Gene Coexpression Network Analysis

The WGCNA uses a stepwise analytical process to interrogate variations in gene coexpression patterns across all samples in the same group (RSV or hRV infection) to identify subsets of coexpressed genes with similar transcriptional patterns together to form a group of transcripts (coexpressed network modules). Because the probability for multiple transcripts to follow a complex pattern of expression across all the samples by chance is low, such sets of genes should constitute coherent and biologically meaningful transcriptional units. The coexpressed network modules that correlate to a clinical trait are assumed to have a coordinated biological function in the clinical condition under study (disease status) (Supplementary Table 1 represents clinical traits of patients at acute phase and early recovery phase) [25]. We focused on groups of transcripts significantly correlated to clinical and laboratory parameters that have been previously implicated in RSV outcomes [24] and that were also differentially expressed during RSV infection.

### Functional Enrichment Analysis

For the biological interpretation of gene lists and WGCNA modules, InnateDB pathway analysis [35] and g:Profiler [36] were used to identify significantly enriched pathways.

### Protein-Protein Interaction Network

To obtain insights into the interaction between DEGs of selected modules, a protein-protein interactions (PPIs) network was constructed by NetworkAnalyst [37] using the IMEx Interactome database [38]. The pipeline of data analysis is summarized in Supplementary Figure 2.

## RESULTS

### Demographics of Microarray Patients

There were no significant differences in sex and number of household members between the 3 selected patient groups as well, compared with the original cohort (Table 1). Patients with single RSV infection were the youngest group. There were no deaths and similar percentages of patients with a history of premature birth between hRV and single-RSV infection groups. It is interesting to note that the RSVco had been significantly under steroids treatment (Table 1).

**Table 1.** Basic Demographic Characteristics of Microarray Patients

Characteristics	RSVsi(n = 38)	RSVco(n = 15)	hRV(n = 30)	Total(N1 = 83)	Total cohort(N2 = 632)
Median age in months (IQR)	6.4 (3.8–11.2)	9.3 (6.8–12.1)	8.15 (3.5–11.95)	7.8 (4.3–11.7)	7 (4–12)
<2 months), n (%)	4 (10.5%)	0 (0%)	2 (6.6%)	6 (7.2%)	41 (6%)
2 to 6 months, n (%)	15 (39.4%)	3 (0.2%)	9 (30%)	27 (32.5%)	277 (44%)
7 to 11 months, n (%)	11 (28.9%)	7 (46.6%)	11 (36.6%)	29 (34.9%)	174 (28%)
12 to 24 months, n (%)	8 (21%)	5 (33.3%)	8 (26.6%)	21 (25.3%)	140 (22%)
Male, n (%)	27 (71%)	10 (66.6%)	19 (63.3%)	56 (67.5%)	430 (68%)
Median number of household members (IQR)#8232;	4 (3.7–5)	5 (3–6)	4 (3–6)	4 (3–5)	4 (3–6)
Premature birth, n (%)	4 (10.5%)	0 (0%)	3 (10%)	7 (8.4%)	54/616 (9%)
Use of steroid, n (%)	3 (7.89%)	3 (20%)	3 (10%)	9 (10.8%)	33 (5%)
Mechanical ventilation, n (%)	0	0	0	0	2 (0.3%)
Day of illness (IQR)#8232;(before admission) (IQR)#8232;	3(3–4)	3(3–4)	2.5 (2–3.25)	3 (2–4)	3 (3–4)
RSV positivity(at discharge or day 7), n (%)	28 (73.7%)	12 (80%)	NA	NA	203/302 (67.2%)
Full recovery, n (%)	31 (81.5%)	10 (66%)	19 (63%)	60 (72.3%)	363/596 (61%)
Death, n (%)	0 (0%)	0 (0%)	0 (0%)	0 (0%)	2 (0.31%)

Abbreviations: hRV, rhinovirus; IQR, interquartile range; N1, microarray population; N2, population of the original cohort; NA, not applicable; RSV, respiratory syncytial virus; RSVco, RSV coinfection group; RSVsi, single-RSV group.

### Common Host Transcriptional Responses Across Nasopharyngeal Swab Specimens and Blood in Respiratory Syncytial Virus and Rhinovirus Infection

Differentially expressed genes were identified in the expression data of NP swabs and blood of RSVsi (182 DEGs in blood and 992 in NP swabs), RSVco (106 DEGs in blood and 479 in NP swabs), and hRV (92 DEGs in blood and 325 in NP swabs) (Table 2 and Supplementary Table 2).

**Table 2.** Number of DEGs (FDR <0.05 and |FC| ≥2) Identified With NP and Blood Samples (Early Recovery Versus Acute Phase)

Type of sample	Blood Samples				NP Samples			
Patient Group	hRV	RSVco	RSVsi	RSV	hRV	RSVco	RSVsi	RSV
Number of samples	60	30	76	106	60	30	76	106
Number of DEGs	92 (0 down, 92 up)	106 (14 down, 92 up)	182 (73 down, 109 up)	155 (38 down, 117 up)	325 (297 down, 28 up)	479 (212 down, 267 up)	992 (622 down, 370 up)	825 (512 down, 313 up)

Abbreviations: DEGs, diffentially expressed genes; FC, fold change; FDR, false discovery rate; hRV, rhinovirus; NP, nasopharyngeal; RSV, respiratory syncytial virus; RSVco, RSV coinfection group; RSVsi, single-RSV group.

**Figure 1.**
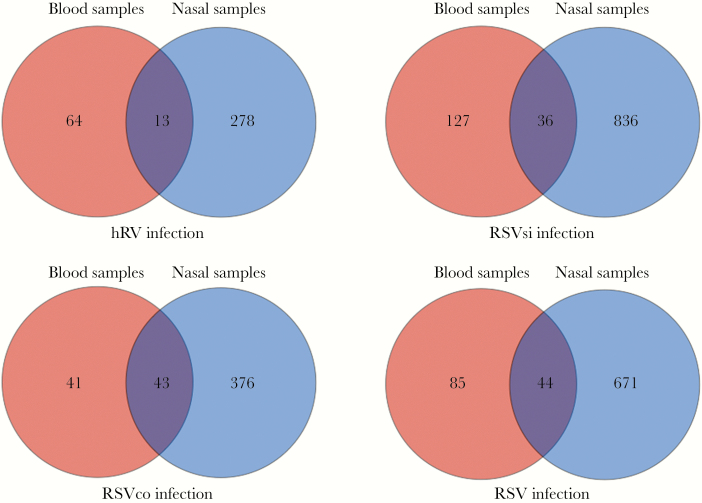
Comparison of differentially expressed genes obtained with nasopharyngeal (NP) swabs and blood samples. Abbreviations: hRV, rhinovirus; RSV, respiratory syncytial virus; RSVco, RSV coinfection group; RSVsi, single-RSV group.

When DEG lists were compared between NP and blood, 36 and 43 overlapping DEGs were observed in RSV single and RSV coinfection, respectively (Figure 1). When we compared DEGs in blood and NP swabs in hRV infection, only 13 overlapping DEGs (of 278 DEGs in NP swabs and of 64 DEGs in blood) were observed (Figure 1), whereas 44 overlapping DEGs (of 671 DEGs in NP swabs and of 85 DEGs in blood) were observed in all RSV infections. Host expression in blood could not mirror all changes in the airway. For RSV infection in general, 52% (44 of 85) of DEGs from peripheral blood reflected 6.5% (44 of 671) of changes in gene expression in the airways versus 20% (13 of 64) and 4.6% (13 of 278), respectively, for hRV infection (Figure 1).

Unsupervised hierarchical clustering heatmaps using DEGs of RSV and hRV in blood and NP swabs confirmed differences in the pattern of gene expression at acute phase and early convalescent phase across 4 patient groups (Supplementary Figure 3). These heatmaps also show more variability between patients infected by the same virus than what is used in the analysis. This advocates for a more thorough analysis of this dataset that lies beyond the scope of this manuscript.

### Functional Annotation and Classification of Differentially Expressed Genes Induced During Respiratory Syncytial Virus and Rhinovirus Infection

**Figure 2. (a):**
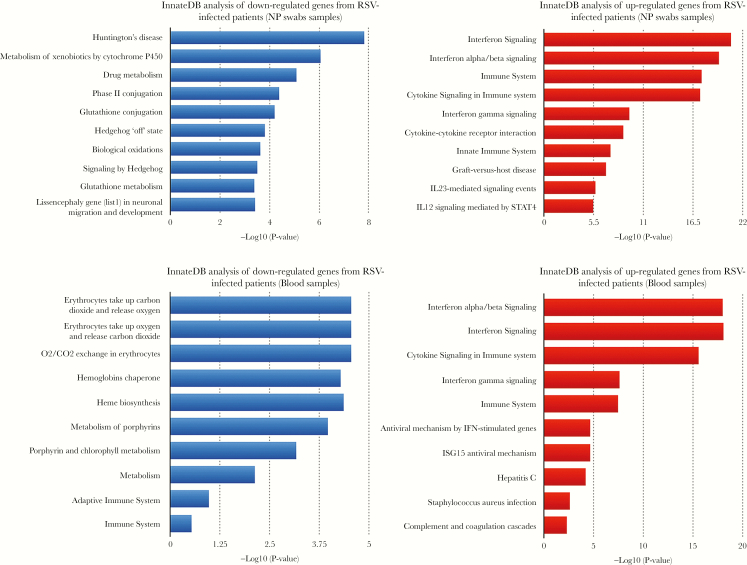

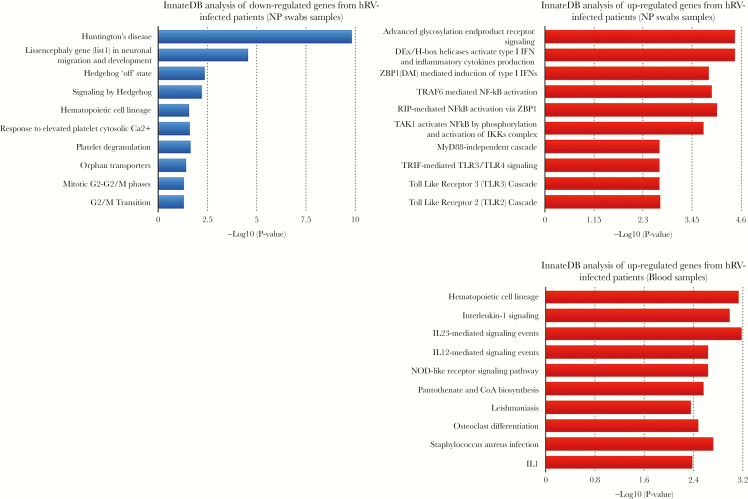
Pathway enriched in differentially expressed genes (DEGs) of nasopharyngeal (NP) swabs and blood in respiratory syncytial virus (RSV) infection using InnateDB. (*b*) Pathway enriched in DEGs of NP swabs and blood in rhinovirus infection. (*a* and *b*) The module-trait relationships with the correlation coefficients and *P* values. The strength of the correlation is colored by different intensities of red (positive correlation) and blue (negative correlation).

By using g:Profiler and InnateDB pathway analysis, we revealed the functional significance of the identified DEGs in all RSV infection cases and in hRV. Innate immune responses, ie, interferon (IFN) signaling (IFN-α/β and IFN-γ signaling), cytokine signaling, and the pathway related to Th17 cell population, ie, interleukin (IL)-23-mediated signaling, were the main pathways enriched from up-regulated genes in both NP swabs and blood of RSV cases. In contrast, the down-regulated genes were involved in specific metabolic processes of different cell types from each compartment (Figure 2a). For hRV infection, all DEGs in blood arrays were up-regulated and enriched in pathways related to (1) innate immune responses, (2) proinflammatory cytokine production (IL-1 signaling pathway), and (3) Th17 cell population (IL-23-mediated signaling) (Figure 2b); however, there were no enriched pathways related to Th17 cell populations in hRV NP arrays.

### Gene Coexpression Network Analysis During Respiratory Syncytial Virus Infection

**Figure 3. (a):**
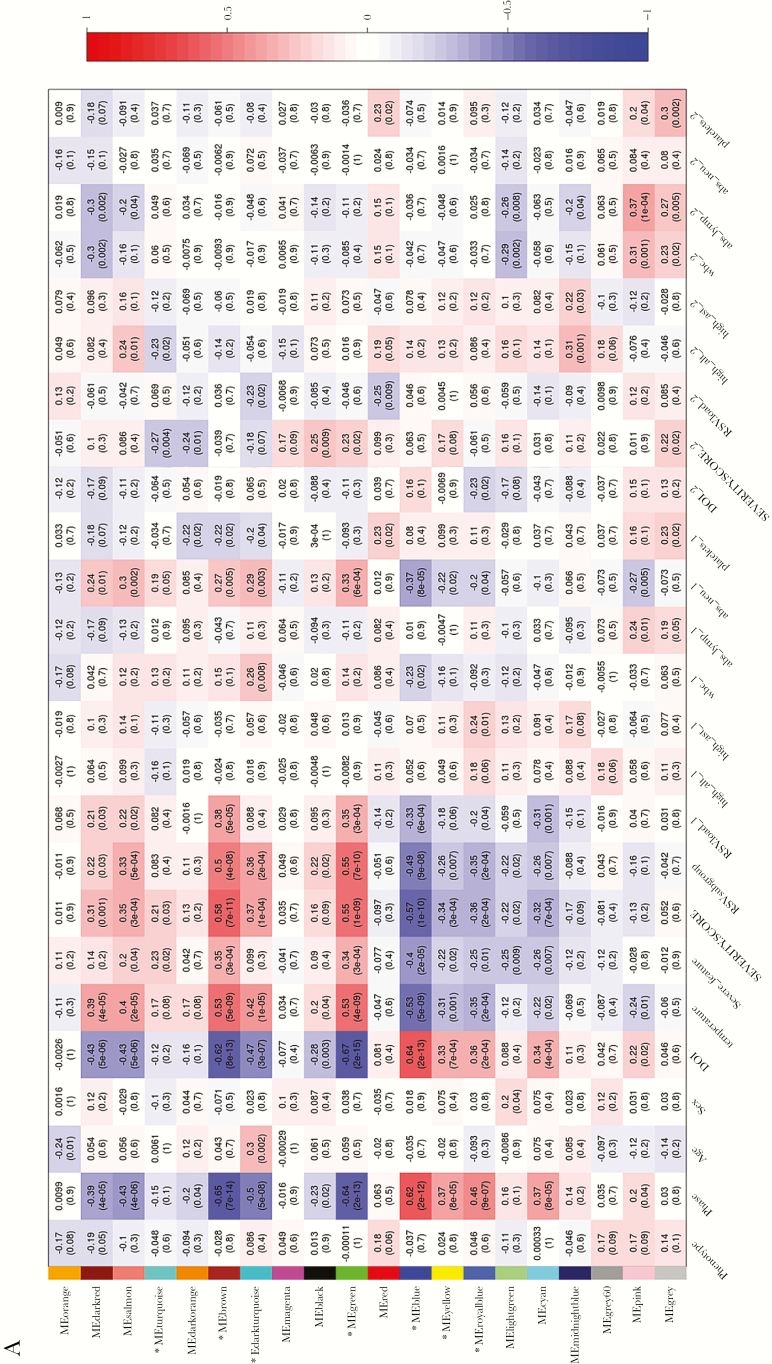

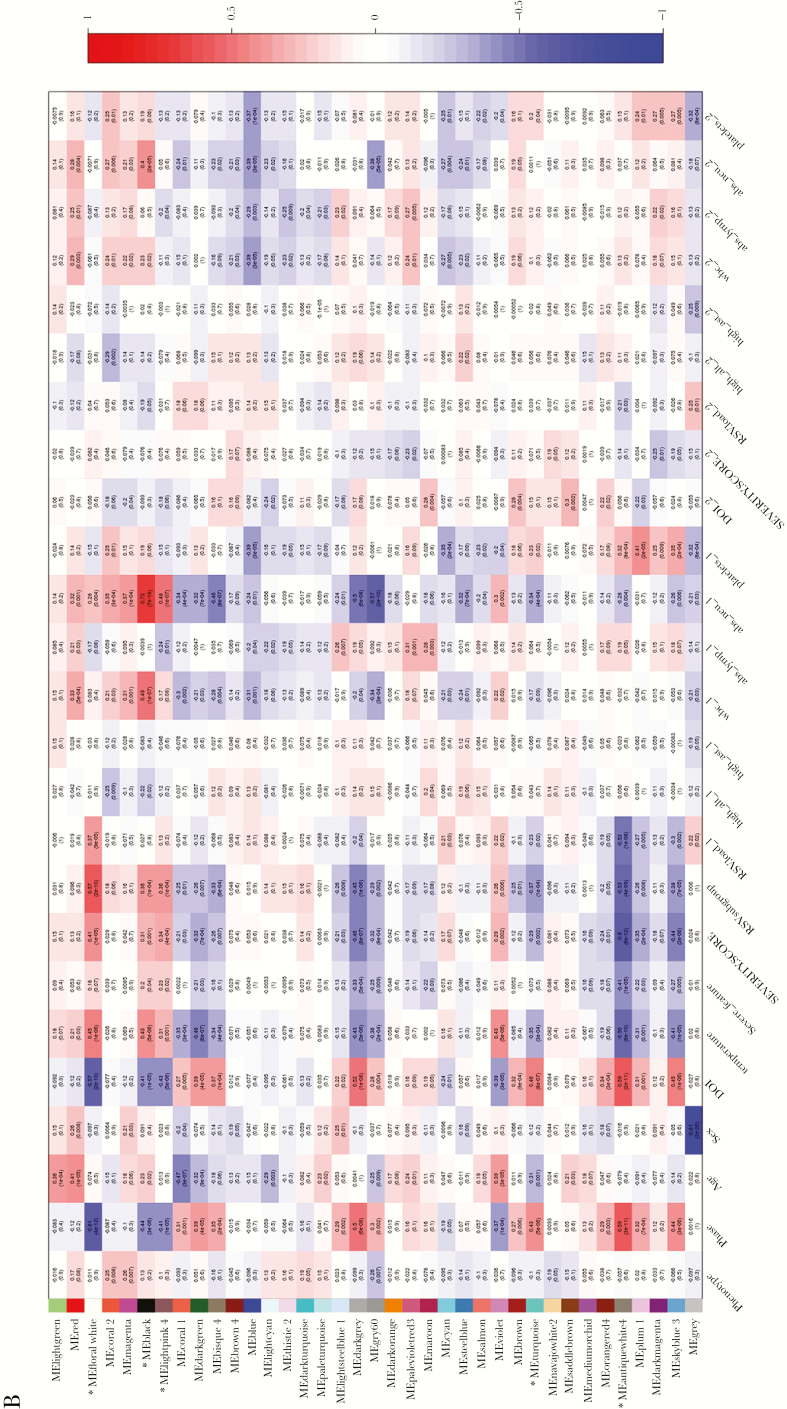
Weighted gene coexpression network analysis (WGCNA) heatmap for gene coexpression network analysis in respiratory syncytial virus (RSV) nasopharyngeal (NP) arrays: identified modules and clinical trait. (*b*) WGCNA heatmap for gene coexpression network analysis in RSV blood arrays: identified modules and clinical trait. Each box represents the module and clinical trait relationships with the correlation coefficients and *P* values. The strength of the correlation is colored by different intensities of red (positive correlation) and blue (negative correlation). The x-axis represents clinical traits and the y-axis represents coexpressed modules. (*) shows the selected modules that were significantly correlated with the course of illness, ie, acute versus early recovery, and with at least 3 clinical traits related to severity, RSV subgroup, and RSV load.

We explored the coexpressed network modules in the RSV host response by using WGCNA, and we identified 20 and 37 coexpressed network modules in NP swabs and blood during RSV infection, respectively (Figure 3a and b). For hRV infection, 22 and 16 coexpressed network modules in NP swabs and blood were identified, respectively (Supplementary Figure 4a and b). We focused on the RSV-coexpressed gene modules that were significantly correlated with the course of illness, ie, acute versus early recovery, and with at least 3 clinical traits related to severity, RSV subgroup, and RSV load. Hence, 7 of 20 modules in NP swabs and 5 of 37 modules in blood were selected and named as selected significantly coexpressed gene modules.

### Identification of Hub Genes Involved in Respiratory Syncytial Virus (RSV) Pathogenesis Through RSV Pathogenesis Networks and Differentially Expressed Genes Identified During RSV Infection

We hypothesized that the common set of genes between the DEGs identified over the course of the RSV infection and the significantly coexpressed gene modules could reveal leading biological pathways and identify key genes related to pathogenesis. Hence, we compared the selected significantly coexpressed gene modules and DEG lists (Supplementary Figure 5). Expression of these modules was either positively correlated with viral load, severity, and blood neutrophil counts or inversely correlated with time, load, and severity (Table 3).

**Table 3.** Characteristics of the Selected Significant Coexpressed Network Modules of Severe and Nonsevere Group in NP Swabs and Blood

Group	Sample Type	WGCNA Module Selected	Correlation of Gene Expression With Phase of Infections and DOI	Correlation of Gene Expression With Severity and RSV Load	Top 3 GO- Functional Enrichment Pathway
Severe	NP	Turquoise	Negative	Positive	Immune response, immune system process, defense response
		Brown	Negative	Positive	Immune response, immune system process, defense response, innate immune response
		Dark-turquoise	Negative	Positive	Immune system process, immune response, T-cell aggregation
		Green	Positive	Negative	Regulation of signal transduction, enzyme- linked receptor protein signaling pathway
	Blood	Floral-white	Negative	Positive	Response to type I interferon, cellular response to type I interferon, innate immune response
		Black	Negative	Positive	Immune system process, response to stress, defense response
		Light-pink4	Negative	Positive	Vesicle-mediated transport, Toll-like receptor 4 signaling pathway, endomembrane system organization
Nonsevere	NP	Blue	Positive	Negative	Cilium morphogenesis, cilium assembly, cilium organization
		Yellow	Positive	Negative	Oxoacid metabolic process, carboxylic acid metabolic process, organic acid metabolic process
		Royal-blue	Positive	Negative	Mitotic cell cycle, cell cycle
	Blood	Turquoise	Positive	Negative	Heme metabolic process, heme biosynthesis process, locomotion
		Antique-white4	Positive	Negative	Endothelial cell-cell adhesion, leukotriene metabolic process

Abbreviations: DOI, day of illness; GO, gene ontology; NP, nasopharyngeal swabs; RSV, respiratory syncytial virus; WGCNA, weighted gene coexpression network analysis.

**Figure 4. (a):**
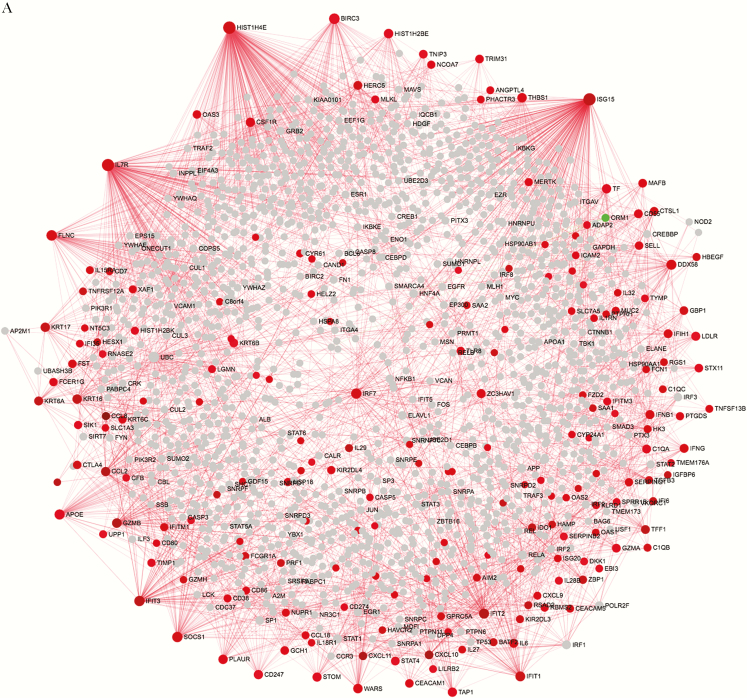

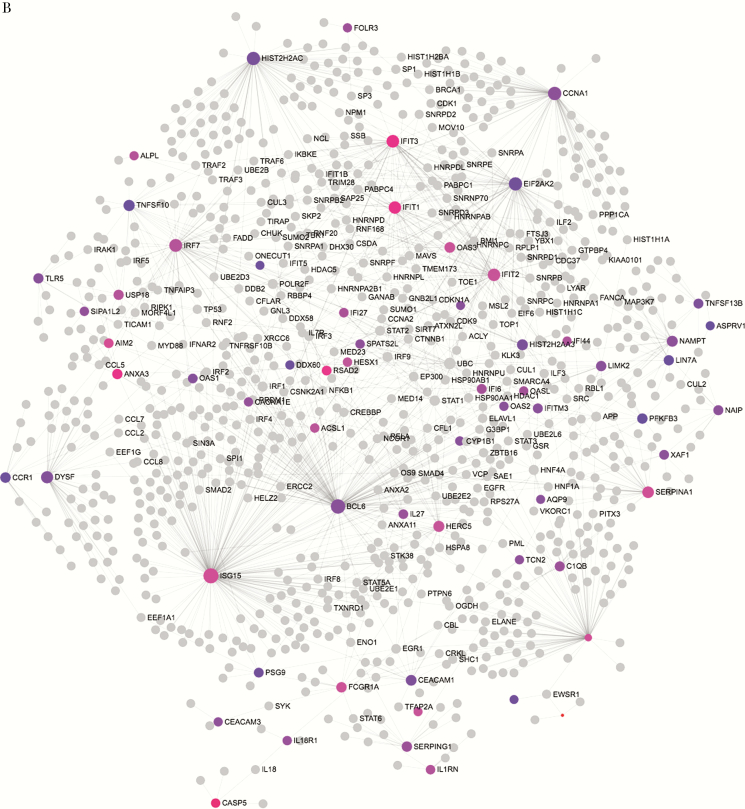
Analysis of acute network in nasopharyngeal (NP). Network analysis using common genes between modules positively correlated with respiratory syncytial virus (RSV) load/severity and those differentially expressed genes (DEGs) identified between acute versus early recovery phase. Red and green nodes represent genes showing increased and decreased expression, respectively. Nodes in gray are direct interaction partners. The size of nodes is proportional to their degree values. (*b*) Analysis of acute network in blood. Network analysis using common genes between modules positively correlated with RSV load/severity and those DEGs identified between acute versus early recovery phase. Nodes in gray are direct interaction partners. The size of nodes is proportional to their degree values, and the color of nodes are proportional to their betweenness centrality values.

The RSV “severe” network (severe NP network) and the RSV “nonsevere” network (nonsevere NP network) in NP arrays were built from 309 genes and 498 genes, respectively (Supplementary Table 3, Figure 4). The RSV severe network in blood arrays (severe blood network) and the RSV nonsevere network were built from 118 genes and 37 genes, respectively (Supplementary Table 6, Figure 4). Genes in the severe and nonsevere networks were either up-regulated or down-regulated, respectively, at RSV acute infection in both sites (NP swabs and blood). However, only gene ORM1 (orosomucoid 1) in severe NP network was down-regulated during RSV acute infection (logFC = −1.2, adjusted *P*-value = 2.7E-03).

To assess the interaction between the DEGs in the selected modules, a PPI network was explored and visualized using NetworkAnalyst. As shown in Figure 4, the severe NP network consisted of 1803 nodes and 2894 edges, and the severe blood network consisted of 1027 nodes and 1323 edges. In the PPI networks, the nodes with a high degree of connection were defined as hub proteins. The topological analysis of these 2 severe networks revealed HIST1H4E (histone cluster 1 H4 family member E) (logFC = 1.56, *P* = 2.8E-10, degree = 231), IL-7 receptor (IL7R) (logFC = 1.46, *P* = 8.8E-10, degree = 199), and IFN-stimulated gene 15 (ISG15) (logFC = 1.99, *P* = 1.7E-09, degree = 188) (in NP samples) as the most significant hubs in the severe NP network; however, ISG15 (logFC = 1.46, *P* = 3.1E-07, degree = 188), B cell lymphoma 6 (BCL6) (logFC = 1.13, *P* = 6.8E-08, degree = 139), and phospholipid scramblase 1 (PLSCR1) (logFC = 1.45, *P* = 4.0E-10, degree = 84) in the severe blood network were hub genes positively associated with both RSV load and severity.

Comparing these two networks with the recently published RSV-host interaction network [39], we found that only 3 DEGs from the severe NP network interacted directly with a viral RSV protein: (1) SLC7A5 (solute carrier family 7 member 5) (logFC = 1.19, *P* = 1.39E-07, degree = 5) interacts with the RSV nonstructural protein 1 (NS1); (2) DDX58 (DExD/H-box helicase 58) (logFC = 1.38; *P* = 3.3E-09, degree = 56) and (3) IFN induced with helicase C domain 1 (IFIH1) (logFC = 1.28; *P* = 6.6E-09, degree = 22) interact directly with the RSV viral N protein.

## DISCUSSION

To our knowledge, this is the first study examining the host gene expression response in children with LRTI with RSV or hRV over the course of infection from 2 compartments, ie, NP swabs and blood samples, representing in situ and systemic responses, respectively. Using an unbiased analytical strategy, we found that RSV infection induced similar pathways in both compartments, whereas for hRV infection, no common pathways were shared between the two compartments. For RSV infection, the overall transcriptional responses in blood were reduced in comparison to the NP compartment, with fewer DEGs and enriched pathways as well as lower fold changes. This finding highlights the localized response to RSV infection and the importance of studying the NP compartment, which has also been previously shown to have a similar gene expression pattern as bronchial epithelium in response to cigarette smoking [40, 41] or in inflammatory responses [42]. This study invites further explorations on the utility of NP specimens as a noninvasive surrogate for bronchial cells in a larger-scale gene expression analysis using the BeadArray platform [20, 23, 43].

Respiratory syncytial virus infection induced a different host response from hRV in terms of local and systemic responses. Furthermore, RSV appears to be the dominant pathogen during coinfections because similar patterns were observed in RSV coinfection cases. Others also found that in bacterial carriages, the response to RSV was also predominant and independent of the microbiome composition in NP [44]. We could not confirm this observation in our study, because we did not attempt to detect bacteria in NP samples. In contrast to hRV infection, we found that RSV induced a large number of chemokines/cytokines and IFN responses that still predominated on day 4 after illness onset. Others observed only a few of these responses in lung tissue from experimental mouse infections on day 5 [13] and after 24-hour post-RSV challenge in adult patients [14]. This highlighted the limitations of experimental infections in animals or human volunteers, because it may not reliably reflect RSV pathogenesis of severe disease in young children. The top 3 of up-regulated DEGs found in our study (chemokine genes [CCL8, CXCL10, and CXCL11] [fold change 16–32]; CD177 [molecule expressed on neutrophil]; otoferlin [OTOF]; and caspase 5 [CASP5; involved in apoptotic cell death] [fold change 4–8]) highlighted the role of neutrophil activation, overexpression of IFN, and other innate immune genes that have previously been reported in RSV infection [22].

Also of note, for RSV infection only, WGCNA showed clear clusters of modules that significantly correlated with severity (Figure 3a and b), whereas for hRV infection, we did not observe this pattern (Supplementary Figure 4). The ISG15 was up-regulated in both blood and NP samples in acute RSV infection and belongs to modules significantly correlated with viral load. The positive correlation of the expression of ISG15 and RSV load has recently been observed in an ex vivo model of respiratory epithelium and confirmed in clinical samples of RSV-infected children [45]. Another gene positively correlating with RSV load and disease severity and significantly up-regulated in the acute phase was BCL6, which was found as a hub gene in blood. B cell lymphoma 6 mediates the development of T follicular helper (Tfh) cells and is an important regulator of Th2 responses. More importantly, it has been suggested that the short-lived, protective, neutralizing antibody response after RSV infection is due to an impaired development of Tfh cells, which are required for plasmablast generation and subsequent antibody formation [46]. B cell lymphoma 6 overexpression has recently been shown for the first time to promote inflammatory responses, but it inhibits antiviral signaling through repressing the IRF7 promoter activity [47]. The down-regulation of the ORM1 gene in the severe NP network suggests a possible correlation between severe RSV infection and uncontrolled inflammatory cytokine production. However, ORM1 is an anti-inflammatory molecule that inhibits neutrophil activation, complement activity, and modulates proinflammatory cytokine secretion by monocytes [48].

Our study has limitations. Our patients were all hospitalized with severe illness and not representative for the entire spectrum of the RSV infection phenotype, and gene expression patterns may not be similar for milder cases managed as outpatients. Only 83/632 patients from the original cohort were selected for the host expression analysis. The selection was based on availability of paired samples and RNA quality for microarray. The demographic characteristics of this subset of patients were not different from the original cohort. On the other end of the spectrum, because mortality was 0%, we also did not have samples from patients who died. A broader spectrum of clinical severity will facilitate the identification of host markers associated with RSV severity. Using steroids can have an impact on the innate immune response. However, the number of patients under this treatment is small (Table 1), and so we were unable to explore this impact. Although our aim was not to identify biomarkers for RSV and hRV infection, a healthy control group would provide us a baseline to correlate the dispersion of the gene expression induced by viral infection and disease severity. Despite the fact that the accuracy of gene expression data from BeadArray platform is highly improved by using multiple probes for each gene, measuring proteins derived from highly expressed genes, ie, cytokines/chemokines, in blood and in prenasal swabs is required to confirm our observations and the potential markers for studying RSV severity.

## CONCLUSIONS

In conclusion, the present study showed that RSV infection induces strong and prolonged innate immune responses with overlap between local and systemic samples, which was not observed in hRV infection. In severe RSV cases, RSV may cause an unbalanced inflammatory response and interact with the BCL6 gene to escape the host immune response.

## Supplementary Data

Supplementary materials are available at *The Journal of Infectious Diseases* online. Consisting of data provided by the authors to benefit the reader, the posted materials are not copyedited and are the sole responsibility of the authors, so questions or comments should be addressed to the corresponding author.

## Supplementary Material

Supplementary_table_1

Supplementary_table_2

Supplementary_table_3

Revision_Supp_Figure1

Revision_Supp_Figure2

Revision_Supp_Figure3

Revision_Supp_Figure4a

Revision_Supp_Figure4b

Revision_Supp_Figure_5

SUPPLEMENT_FIGURE_LEGEND

## Notes

Presented in part: Australian Respiratory Virology Meeting, December 4, 2015, University of Canberra; RSV16, September 28–October 1, 2016 Argentina (http://www.rsv16.org/); EASyM: 1st Conference of the European Association of Systems Medicine, October 26–28, 2016, Berlin (https://www.easym.eu/); IRCI2017, February 13–15, 2017, Lyon (http://irci2017.insight-outside.fr/).

## Notes

***Acknowledgments.*** The study would not have been possible without excellent support from clinicians at the Paediatric Intensive Care Unit, Emergency Department, and the Paediatric Respiratory Wards at the Children’s Hospitals 1 and 2, Ho Chi Minh City, Vietnam. Special thanks to nurses Hoang thi Minh Tu, Nguyen thi Hong Ngoc, Nguyen Viet Truong, Nguyen thi Ngoc Ha, Nguyen thi Thanh Nha, Le thi Kim Loan, Huynh thi Phuong Thao, and Tran thi Tuyet Nhung at Children’s Hospitals 1 and 2 for help with sample collection. We acknowledge staff members at Data Management at Oxford University Clinical Research Unit (OUCRU) for entering data, Vo Nhi Ha and Nguyen thi Thanh Thuy and the Clinical Trials Unit at OUCRU and Children’s Hospital 1 and 2 for coordinating the study, and Ms Vo Thuy and Ms Thuc Doan for helping receiving and storing samples. Many thanks to Hoang Truong Long and Ling Ling (Infectious Disease Group in Genome Institute of Singapore) for their introduction of basic microarray analysis and the weighted gene coexpression network analysis approach.

***Disclaimer.*** The funders had no role in study design, data collection and analysis, decision to publish, or preparation of the manuscript.

***Financial support.*** This work was funded by Singapore’s Agency for Science, Technology and Research, the PREPARE Consortium (FP7 no. 602525), the eTRIKS Consortium (IMI no. 115446), and Wellcome Trust of Great Britain Grant (077078/Z/05/Z).

***Potential conflicts of interest.*** All authors: No reported conflicts of interest. All authors have submitted the ICMJE Form for Disclosure of Potential Conflicts of Interest.
