# Supplementary figures and images for: Host Transcription Profile in Nasal Epithelium and Whole Blood of Hospitalized Children Under 2 Years of Age With Respiratory Syncytial Virus Infection
Source: J Infect Dis. 2017 Sep 27;217(1):134–46. doi: 10.1093/infdis/jix519 (PMC5853303; doi:10.1093/infdis/jix519)

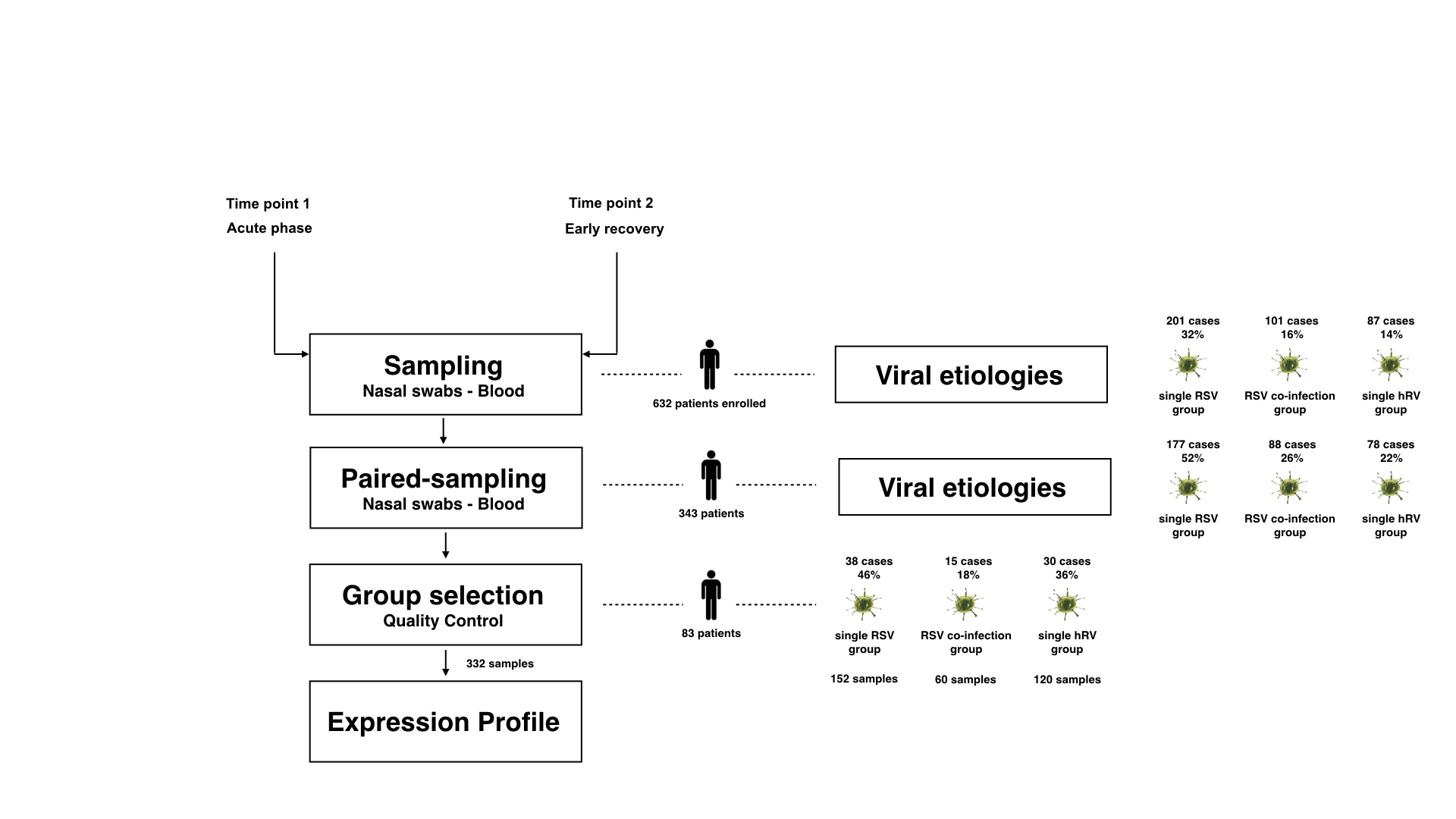

Supplement: Revision_Supp_Figure1 [file jix519_suppl_revision_supp_figure1.png]

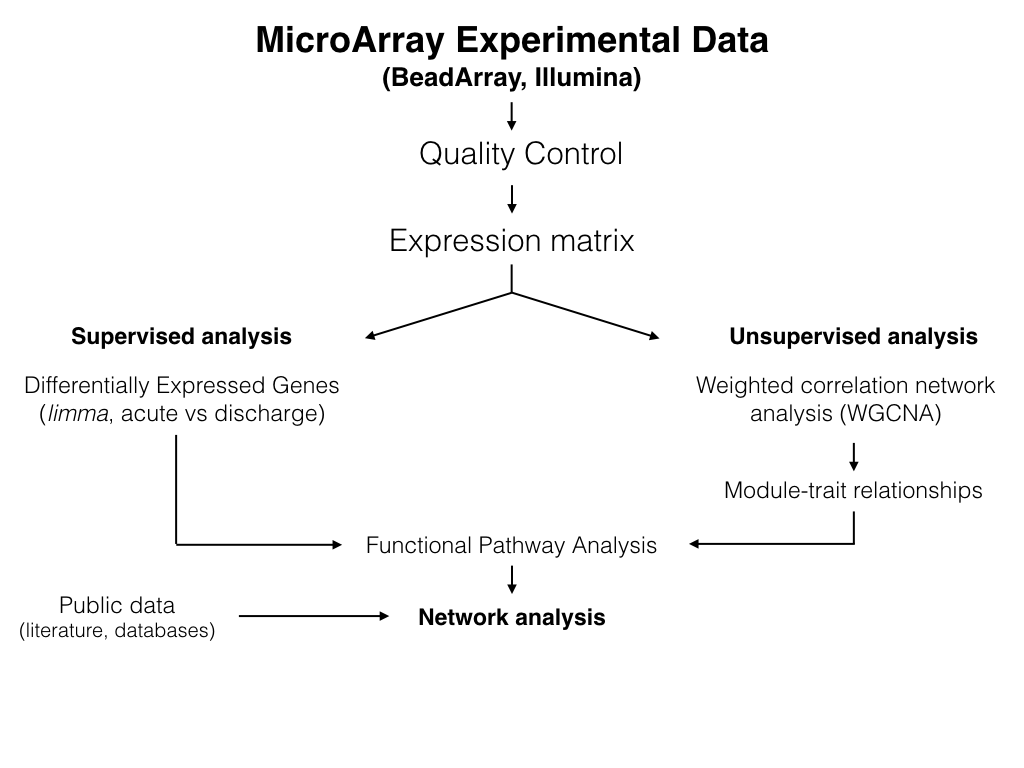

Supplement: Revision_Supp_Figure2 [file jix519_suppl_revision_supp_figure2.png]

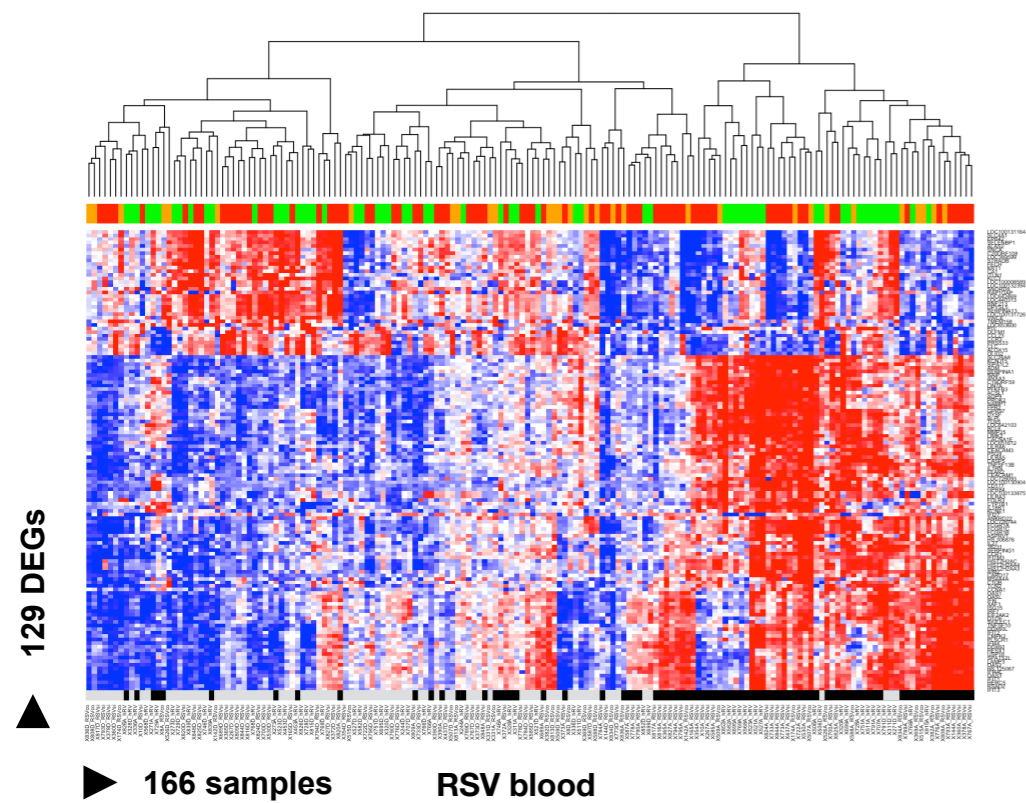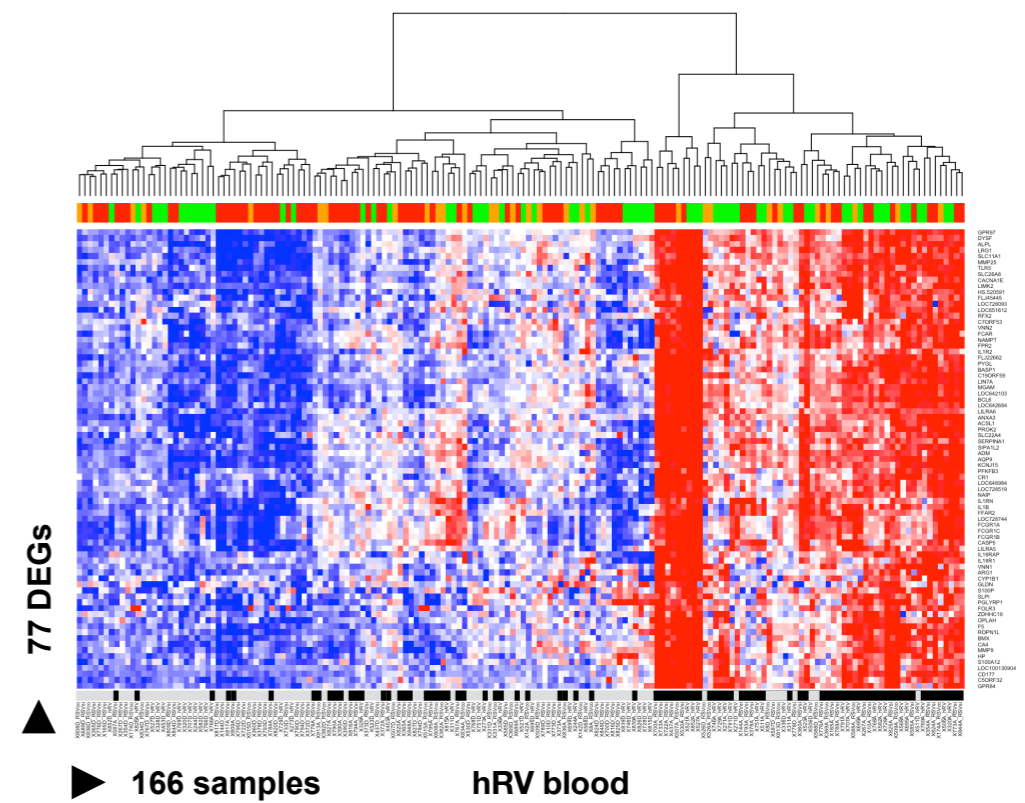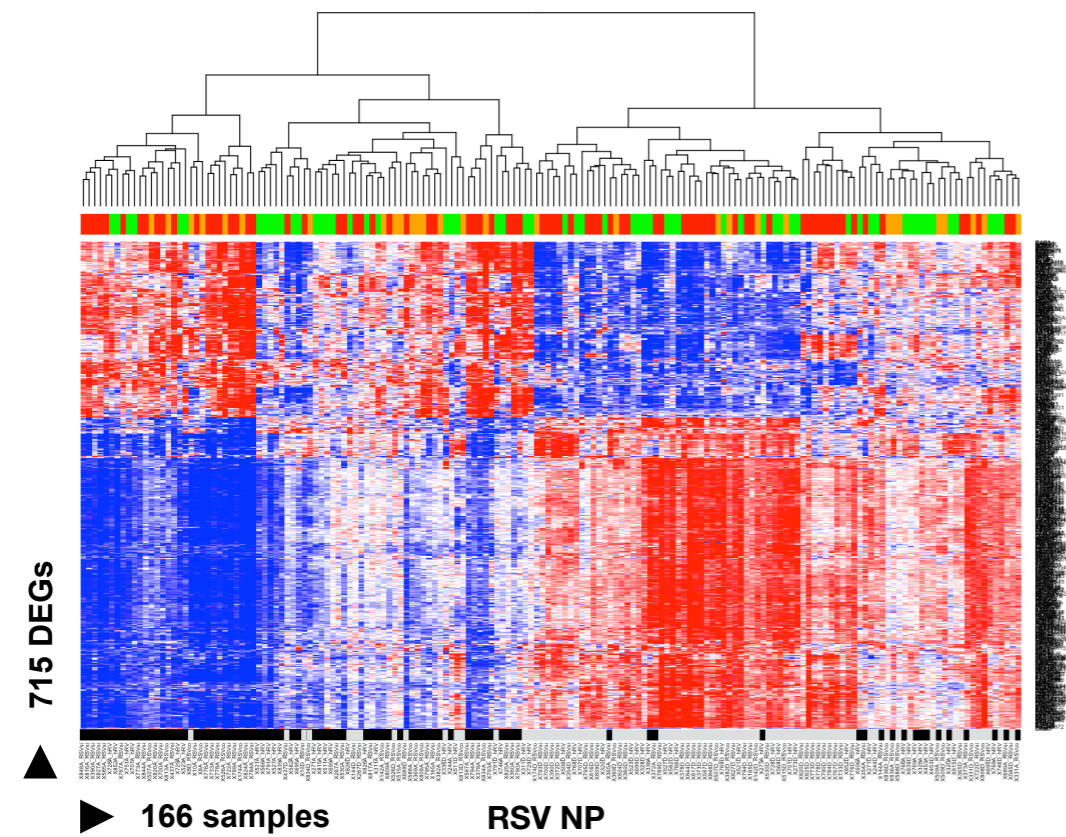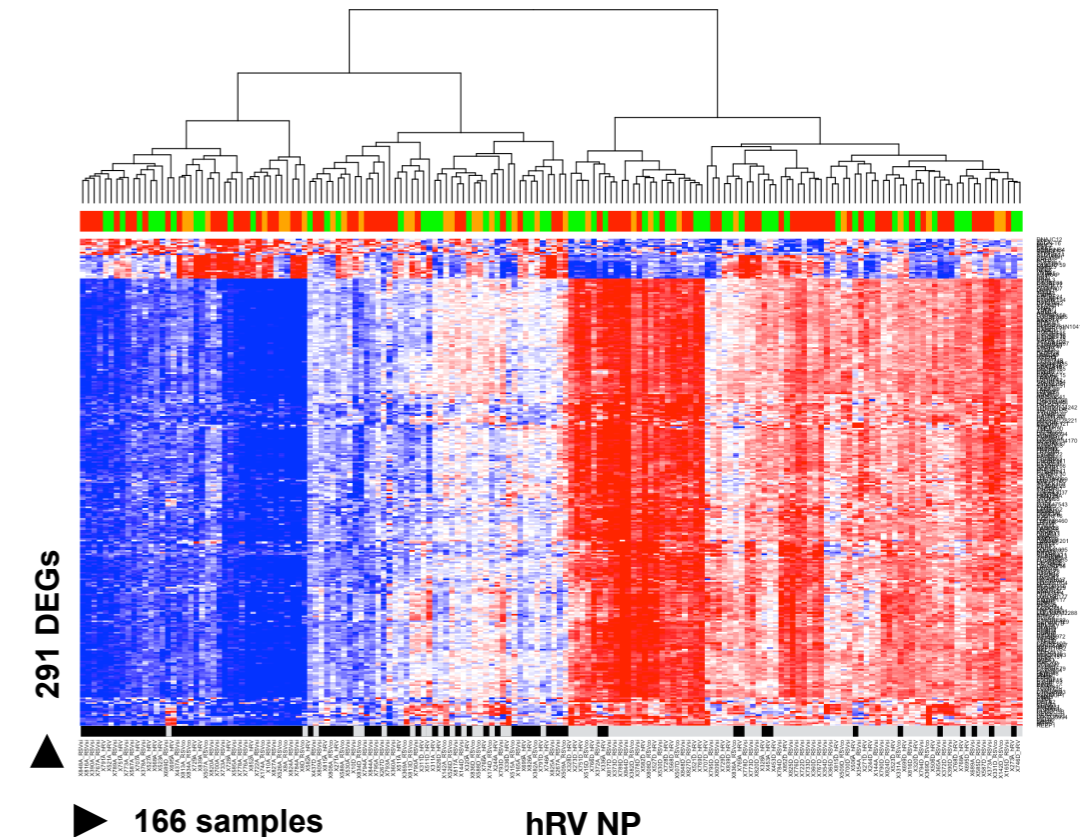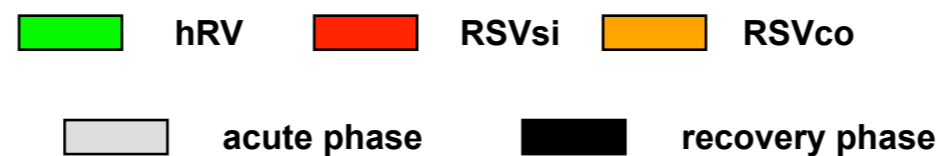

Supplement: Revision_Supp_Figure3 [file jix519_suppl_revision_supp_figure3.pdf]

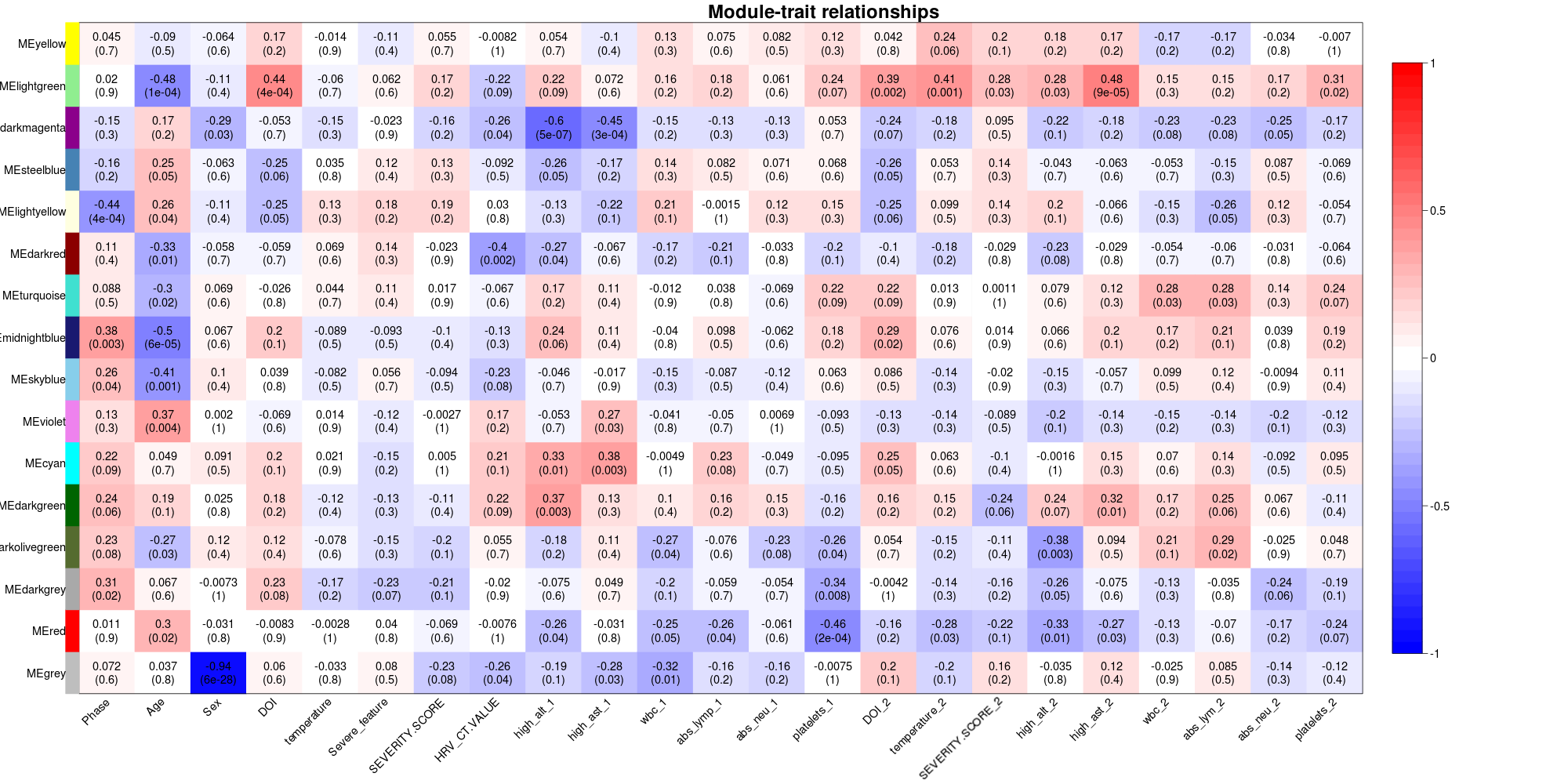

Supplement: Revision_Supp_Figure4a [file jix519_suppl_revision_supp_figure4a.png]

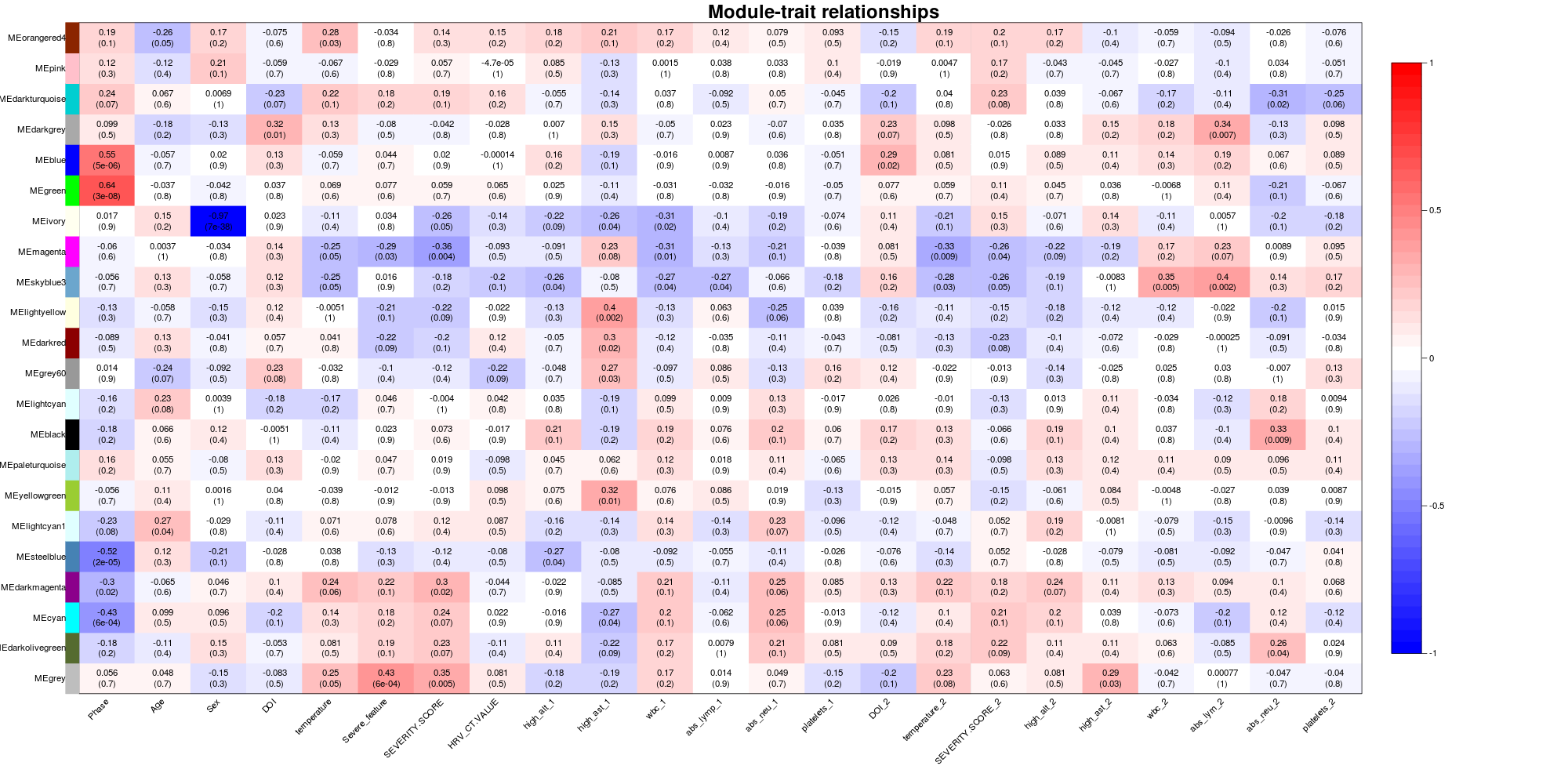

Supplement: Revision_Supp_Figure4b [file jix519_suppl_revision_supp_figure4b.png]

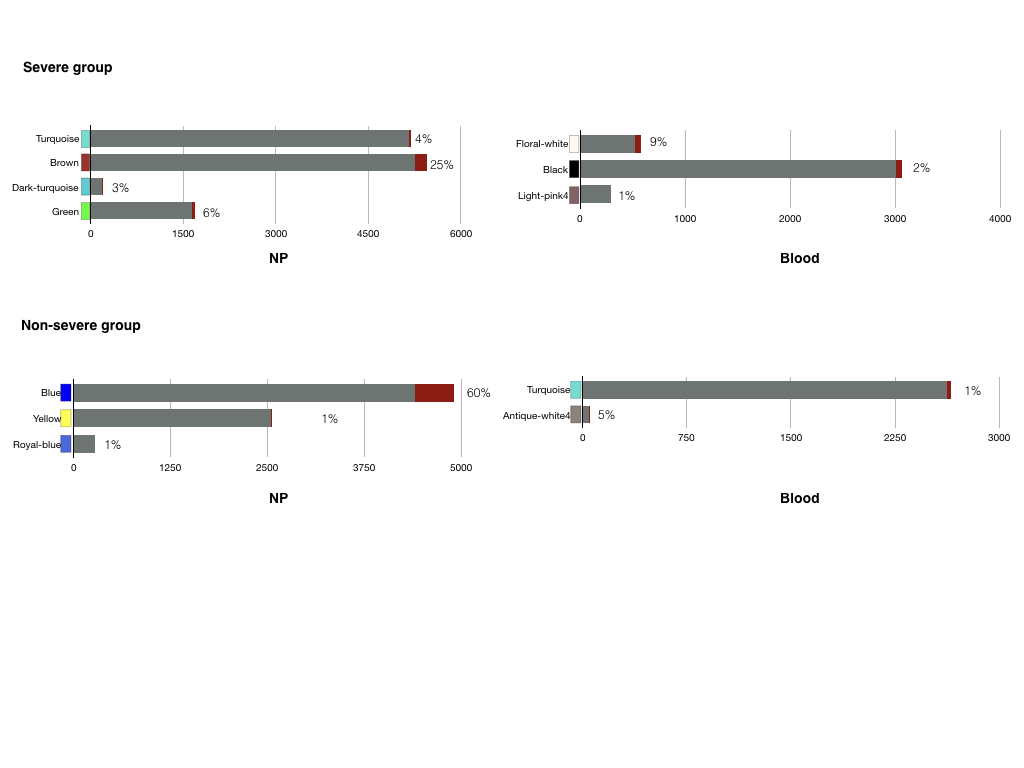

Supplement: Revision_Supp_Figure_5 [file jix519_suppl_revision_supp_figure_5.png]
