# Supplementary material for: Host Transcription Profile in Nasal Epithelium and Whole Blood of Hospitalized Children Under 2 Years of Age With Respiratory Syncytial Virus Infection
Source: J Infect Dis. 2017 Sep 27;217(1):134–46. doi: 10.1093/infdis/jix519 (PMC5853303; doi:10.1093/infdis/jix519)
Supplement: SUPPLEMENT_FIGURE_LEGEND [file jix519_suppl_supplement_figure_legend.docx]

**SUPPLEMENT FIGURE LEGEND:**

**Supp. Figure 1-** Patient enrolment and sampling

**Supp. Figure 2** – Pipeline of data analysis.

**Supp. Figure 3 –** Unsupervised hierarchical clustering heatmaps of differentially expressed genes across 3 patient groups *i.e* single-RSV infection, RSV co-infection and hRV infection

Legend Supp.Figure 3:

*The heatmaps were built on the gene list derived from the differentially expressed genes identified in NP or blood (acute vs recovery phase) for RSV and hRV infection, respectively.*

*Three patients groups are indicated by colored rectangles: red for single-RSV infection (RSVsi), orange for RSV co-infection (RSVco) and green for single rhinovirus infection (hRV).*

*Acute or recovery phase sampling is indicated under the heatmaps by grey and black rectangles respectively.*

*Normalized expression levels are indicated as overexpressed (red) or underexpressed (blue).*

*The x-axis represents samples and the y-axis represents number of genes used for the heatmaps. For differentially expressed probes pointing to the same official gene name, we calculated the median of their normalized expression.*

**Supp.Figure 4** - WGCNA heatmap for gene co-expression network analysis in hRV

**Supp.Figure 4a -** WGCNA heatmap for gene co-expression network analysis in hRV NP arrays: identified modules and clinical trait

**Supp.Figure 4b** - WGCNA heatmap for gene co-expression network analysis in hRV blood arrays: identified modules and clinical trait

Legend Supp.Figure 4: *Each box represents the module and clinical trait relationships with the correlation coefficients and p-values. The strength of the correlation is colored by different intensities of red (positive correlation) and blue (negative correlation). The x-axis represents clinical traits and the y-axis represents co-expressed modules.*

**Supp. Figure 5** - Percentage of differentially expressed genes (DEGs) in the co-expressed network modules related to severity and viral load in NP and blood samples

Legend Supp. Figure 5*: 7 selected significantly co-expressed gene modules in NP have gene size ranging from 281 to 5454 genes, while 5 selected significantly co-expressed gene modules in blood have 54 to 3060 genes. The proportion of DEGs within each module is indicated in red on the bar plots.*
